# Novel Perspectives in Pseudomyxoma Peritonei Treatment

**DOI:** 10.3390/cancers13235965

**Published:** 2021-11-27

**Authors:** Antonio Sommariva, Marco Tonello, Giulia Rigotto, Nayana Lazzari, Pierluigi Pilati, Maria Luisa Calabrò

**Affiliations:** 1 Surgical Oncology of the Esophagus and Digestive Tract, Veneto Institute of Oncology IOV-IRCCS, 35100 Padua, Italy; marco.tonello@iov.veneto.it (M.T.); pierluigi.pilati@iov.veneto.it (P.P.); 2 Immunology and Molecular Oncology, Veneto Institute of Oncology IOV-IRCCS, 35100 Padua, Italy; giulia.rigotto@iov.veneto.it (G.R.); nayana.lazzari@iov.veneto.it (N.L.); luisella.calabro@iov.veneto.it (M.L.C.)

**Keywords:** Pseudomyxoma Peritonei, cytoreductive surgery, HIPEC, mucin, peritoneal metastases

## Abstract

**Simple Summary:**

Pseudomyxoma Peritonei (PMP) represents a rare entity which greatly benefits from Cytoreductive Surgery (CRS) associated with Hyperthermic Intraperitoneal Chemotherapy (HIPEC). In fact, CRS-HIPEC represents the treatment with potential chances of cure and long-term disease control of patients affected by PMP. This therapeutic strategy should be performed in referral centers, where a consolidated know-how of this locoregional treatment and a multidisciplinary approach are available. CRS-HIPEC provides excellent results for PMP patients in terms of postoperative outcome, overall and disease-free survival, and quality of life. However, in patients with an extensive or recurrent disease, few therapeutic opportunities are available. This review is focused on the most recent clinical evidence and provides a better understanding of the molecular prognostic factors and potential therapeutic targets in this rare malignancy.

**Abstract:**

Pseudomyxoma Peritonei (PMP) is an anatomo-clinical condition characterized by the implantation of neoplastic cells on peritoneal surfaces with the production of a large amount of mucin. The rarity of the disease precludes the evaluation of treatment strategies within randomized controlled trials. Cytoreductive Surgery (CRS) combined with Hyperthermic Intraperitoneal Chemotherapy (HIPEC) has proven to be the only therapeutic option with potential chances of cure and long-term disease control. The present review discusses the epidemiology, pathogenesis, clinical presentation and treatment of PMP, focusing on the molecular factors involved in tumor progression and mucin production that could be used, in the upcoming future, to improve patient selection for surgery and to expand the therapeutic armamentarium.

## 1. Introduction

**Figure 1:**
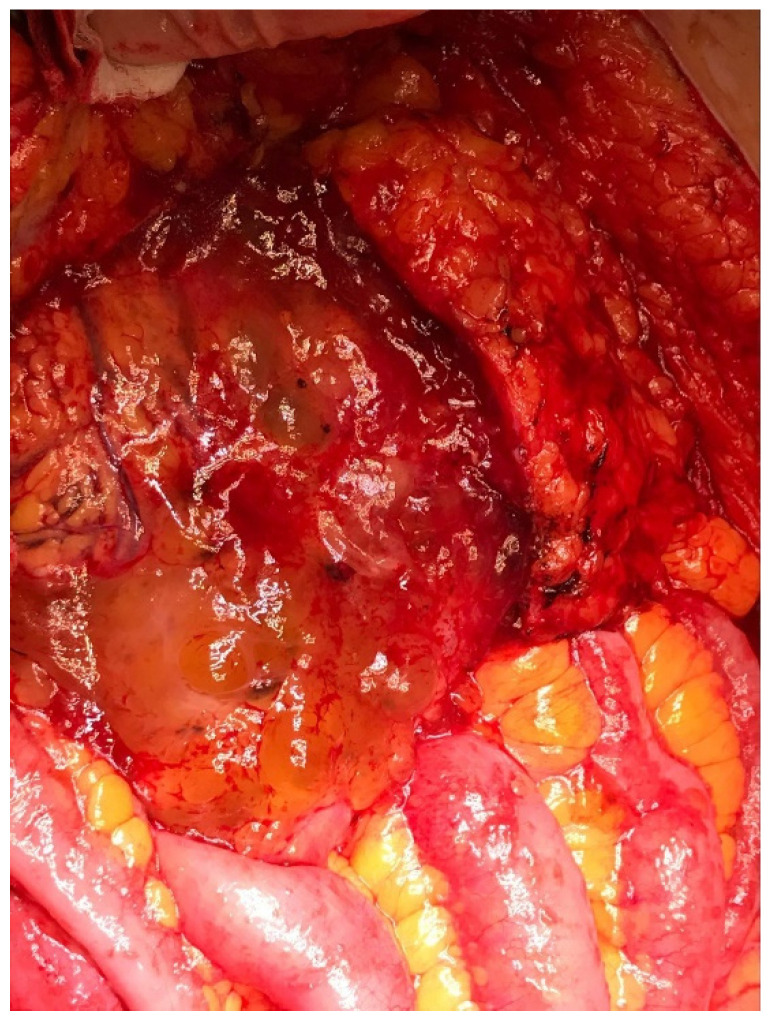
Intraoperative view of Pseudomyxoma Peritonei.

Pseudomyxoma Peritonei (PMP) is a peculiar type of peritoneal metastases mainly determined by an intraperitoneal dissemination of appendiceal mucinous tumors. The clinical course is characterized by the implantation of neoplastic cells on peritoneal surfaces with the production of mucin throughout the abdominal cavity, such as mucinous ascites (Figure 1).

This clinical picture was first described in 1884 by Werth [1]. The relative absence of symptoms in the early phases of the disease progressively favors the accumulation of large volumes of mucin inside the abdomen, finally resulting in huge abdominal enlargement, pain and malnutrition. Compression on visceral organs and the inflammatory and fibrotic reaction of mesothelium determines over time intestinal obstruction, which is a fatal complication of untreated or recurrent PMP. Because of its indolent course, historical treatment of PMP consists of serial non-radical debulking surgeries, essentially aimed at controlling symptoms and complications [2]. However, patients frequently recur and become over time inoperable and die of malnutrition or complications after salvage surgery. In the early 1990s, a substantial improvement in the treatment of PMP was obtained after the introduction of a more aggressive surgical approach (Cytoreductive Surgery, CRS) associated with Hyperthermic Intraperitoneal Chemotherapy (HIPEC). Nowadays, CRS-HIPEC represents the only treatment with potential chances of cure and long-term disease control of patients affected by PMP [3]. When performed in referral centers, where a consolidated know-how of this locoregional treatment and a multidisciplinary approach are available, CRS-HIPEC provides excellent results in terms of postoperative outcome, overall survival (OS) and disease-free survival and quality of life [4]. In a retrospective analysis of 1924 patients treated with surgery, CRS-HIPEC provided a significant better survival outcome in comparison to CRS alone (5-year OS: 57.8% vs. 46.2%, respectively). CRS-HIPEC was associated with a 90-day mortality of 4.2% and a severe morbidity rate of 32% [4]. Histopathologic features of PMP (low- vs. high-grade) and the completeness of cytoreduction are the main factors associated with survival and disease-free outcome [5]. CRS-HIPEC represents an overly complex procedure [6]. Cytoreduction combines multiple peritonectomies (parietal, diaphragmatic and pelvic) and visceral resections (gastrointestinal, hysterectomy and splenectomy) with the ‘‘electro evaporation’’ of unresectable nodules (i.e., on Glisson’s capsule, small bowel surface and mesenteries). The main surgical goal is to obtain optimal cytoreduction with no macroscopic residual disease, considering the strong correlation between the completeness of cytoreduction and the outcome. HIPEC is started immediately after the completion of surgery and is obtained by inserting intraperitoneal catheters in the abdominal cavity connected with a pump supplied by a heater and a heat exchanger [7]. The duration of perfusion combined with the optimal abdominal temperature depends on the protocol used and no systematic experimental study has been carried out to identify the most effective perfusion protocol. Most centers use an Oxaliplatin- or Mitomycin C (MMC)-based chemotherapy, reporting a temperature range of 41.5–43.0 °C with a perfusion time from 30 to 90 min (Table 1). A wide variability on the type, dosage, carrier solution and duration of HIPEC is reported. A recent retrospective review showed a survival advantage in patients treated with Oxaliplatin, 5-Fluorouracil/Leucovorin and with MMC/Cisplatin HIPEC regimens [4], in addition to a higher morbidity profile of MMC-based regimens.

The scanty safety associated with CRS-HIPEC has represented in the past one of the main concerns regarding this locoregional approach. The refinement of anesthesiologic protocols and a better patient’s selection make CRS-HIPEC a safe procedure, associated with morbidity and mortality rates similar or lower to those of other major abdominal surgical procedures [8].

Though CRS-HIPEC is considered the gold standard of treatment for PMP, several questions still remain unanswered and highly debated. In this perspective, clinical research in PMP is difficult because of the rarity of the disease, and randomized controlled trials in humans are practically impossible to arrange [9]. Moreover, the paucity of the cellular component of PMP hinders a proper cell isolation and culture, necessary steps for research on molecular mechanisms of tumor growth and mucin production. From therapeutic perspectives, every effort should be done for ameliorating the outcome of patients selected for CRS-HIPEC, mainly through the standardization of the procedure and quality control. Moreover, the diagnostic pathway should be better defined in the early phase of the disease, including second-look strategies in patients operated outside referral centers. Patients not suitable for CRS-HIPEC for advanced disease, poor clinical condition and recurrent disease unable to be surgically treated have an extremely limited therapeutic armamentarium. Standard chemotherapy or targeted therapy have a limited role in symptoms and disease control, and the identification of new molecular targets is needed to extend the therapeutic opportunities of PMP patients. Basic research of the biomolecular factors involved in PMP tumor growth and mucin production could, in the future, improve outcomes after optimal radical surgery, as well as reduce symptoms and complications in patients with unresectable disease. Table 2 summarizes the therapeutic approaches and their recommended use in PMP patients.

The aim of this study is to review the more controversial aspects of PMP treatment with a focused perspective on the ongoing clinical and molecular research in this field.

## 2. Standardization of PMP Treatment

The standardization of surgical treatment is an essential step, strictly correlated with the goal of a proper quality control of the treatment of PMP patients. The rarity of the disease precluded the design of prospective trials, and most of the evidence came from a retrospective analysis of single centers’ experience or data registry. For this reason, national guidelines do not give a clear indication of how PMP has to be treated and followed, exclusively suggesting to send the patients to a referral center. The recent consensus based on the expert opinion promoted by the Peritoneal Surface Oncology Group International (PSOGI) [10] tried to define the best algorithm for diagnosis, treatment and follow-up. The PSOGI survey involved 80 worldwide experts, who participated during three voting rounds (Delphy methodology) to reach a consensus on 69 recommendations regarding PMP and appendiceal tumors. The PSOGI consensus found a substantial agreement on the clinical and diagnostic aspects of PMP management. A preoperative work-up, including CT scan, markers (CEA, Ca 19-9 and Ca 12-5) and colonoscopy, was considered indicated by the vast majority (>95%) of panelists. Laparoscopy to obtain tissue diagnosis and confirm resectability can also be considered. MRI was considered as a complementary tool for staging, while the role of PET-CT remained controversial and recommended by half of the panelists only. A full agreement was found in performing CRS-HIPEC in patients fit for surgery when the disease is resectable and referred to a peritoneal cancer center.

A crucial point in the perspective of standardization is to define a pathological classification able to stratify the patient’s prognosis and allowing a more tailored approach for treatment and follow-up. An almost absolute consensus (98.2%) on the classification previously proposed by pathologists referring to the PSOGI consortium has been obtained [11]. Three categories of PMP have been recognized: low-grade, high-grade and high-grade with signet ring cells. The presence of acellular mucin has been classified separately. In parallel, appendiceal mucinous neoplasm causing PMP have been classified with the same criteria: low-grade appendiceal mucinous neoplasm (LAMN) and high-grade appendiceal mucinous neoplasm (HGMN). Appendiceal mucinous tumors with clear infiltrative patterns should be reported as mucinous adenocarcinoma with or without signet ring features. In 2019, a classification of appendiceal neoplasm was developed by the World Health Organization (WHO) on the PSOGI consensus [12]. The TNM classification differs mainly for LAMN, as pT1-2 stages (submucosa-muscolaris propria invasion) are lacking and lesions confined in the submucosa are classified as in situ (pTis). For distant metastasis, pM1a is defined as the presence of acellular mucin only, if the presence of mucinous epithelium in the peritoneal cavity is classified as M1b. The WHO grading of mucinous appendiceal neoplasm defines as G1 the LAMN, G2 the HGMN and mucinous adenocarcinoma and G3 mucinous adenocarcinoma with signet ring cells. The same grading is used in the presence of PMP. Although the PSOGI classification is extensively adopted by most centers performing CRS-HIPEC, its prognostic role is unclear and no prospective data are available to verify whether this three-tier system might provide a better stratification than the current 2019 WHO classification. Further studies in larger prospective series are needed [13].

## 3. Quality Assurance

Measuring the quality of surgical care represents a research field in continuous evolution, as standardization and high quality of surgery in oncology guarantee better outcomes for patients, as well as reduced costs and better resources allocation for the health care system, especially in a highly complex and risky procedure such as CRS-HIPEC [14,15]. In this perspective, the centralization of the procedures in centers with expertise in PMP treatment is recommended as one of the main factors for the improvement of quality of the treatment, as hospital volume is a parameter frequently used for high-risk and complex procedures. Several reports indicate that the caseload of the center may have a direct effect on the improved outcomes after CRS-HIPEC [16]. However, factors and processes potentially leading to a better outcome in high-volume centers are largely unknown, and therefore, they cannot be readily modified/readjusted to improve quality after CRS-HIPEC.

Early recurrence represents an interesting quality indicator because it reflects the whole process of the diagnosis, treatment and follow-up of PMP patients treated with CRS-HIPEC. A retrospective study carried out in 2451 PMP patients treated by 47 surgeons in 33 referral centers clearly showed that not only biological factors but also surgeons’ background and institutional organization might influence the results [17]. This retrospective analysis, which considered early recurrence (<2 years) of PMP as a quality indicator after CRS-HIPEC, showed that center volume is significantly related to early oncological failure. This study confirmed that the annual caseload of less than 60 cases (any histology) is an independent predictor of oncological failure. Moreover, the proportion of PMP patients treated in a referral center, in comparison with other histologies, independently influences the rate of early recurrence. This means that the quality of treatment of PMP patients encompasses not only the technical aspects of peritoneal cancer surgery, but also a wider range of factors based on the deep knowledge of the biology and natural history of this rare tumor. Defining the benchmark value of these quality parameters could, in the upcoming future, guide health care providers to reduce costs and allow a better resource allocation. Considering the annual incidence of PMP, these benchmark values are likely to define an ideal relationship between number of inhabitants and number of centers where PMP treatment can be offered with adequate quality levels. Further research on relevant, reliable, valid and easily usable indicators is needed to continuously monitor PMP treatment through properly designed quality assurance programs at institutional and national levels [16].

## 4. New Surgical Approaches

### 4.1. Second-Look Strategy after Diagnosis of Appendiceal Mucinous Tumor

The management of patients with unexpected mucinous appendiceal tumors is frequently problematic as the majority of patients are treated for a suspected acute appendicitis outside peritoneal cancer centers. Frequently, intraoperative findings are not well-described as mucin can get mistaken for purulent abdominal contamination. The pre-referral management of these tumors is not standardized and the management by surgeons’ experts in peritoneal cancer management is also variable [18]. The most controversial point deals with the risk for progression to PMP in these patients and whether a second-look strategy after negative radiological staging should be considered for early diagnosis and treatment. In a large population-based study, metachronous PMP after appendectomy for appendiceal tumors is estimated to occur in more than 20% of cases, with a median latency time of 2 years and up to 10 years [19]. The unexpected finding of mucinous neoplasms during surgery for appendicitis occurs also when surgery has been planned in the context of an interval appendectomy strategy after conservative management. Following interval appendectomy, the incidence of mucinous neoplasm is even higher (up to 29.6%) in a single institution experience [20]. However, in a retrospective analysis of patients with LAMN treated outside a referral institution but evaluated by a peritoneal cancer center according to an internal protocol of pathology review and follow-up (median: 58 months), the occurrence of PMP was only 2% [21]. These data clearly suggest that each patient with a diagnosis of mucinous appendiceal tumor should at least be further evaluated in a referral center. Mucin and neoplastic cells in the appendiceal wall or periappendiceal tissue and peritoneal cavity are recognized as risk factors for PMP progression [22,23]. Considering that a low tumor load has a significant prognostic impact in PMP, early detection and treatment of mucinous nodules could allow for the best chance of definitive cure of these patients. Based on this rationale, a study investigating a systematic evaluation with CT scan and exploratory laparoscopy after LAMN diagnosis revealed peritoneal disease in 23% of patients [24]. Moreover, patients with negative laparoscopy remained relapse-free after a median follow-up of 50 months [24]. A prospective study confirmed that despite a negative CT scan, a significant percentage of patients with high-risk LAMN have acellular mucin (23.6%), mucin with epithelium (3.5%) or residual appendix tumor present at laparoscopic second-look [25]. Therefore, a systematic evaluation by an experienced center in presence of histologically confirmed extra-appendicular spread after primary surgery should be considered. In this setting, laparoscopy represents the ideal means for performing CRS and HIPEC delivery. Data from the PSOGI registry show that laparoscopic CRS-HIPEC is safe and feasible in highly selected patients with limited peritoneal disease when performed at experienced centers [26]. Further prospective studies with a longer follow-up will clarify if this second-look strategy in high-risk LAMN patients can modify the natural history of the disease.

### 4.2. Surgery for Unresectable PMP

Debulking surgery represents a valuable option in patients not fit for an extended CRS, or with high tumor burden/unresectable disease. Maximal tumor debulking should be tailored to the clinical condition, avoiding cytoreductive procedures in the upper abdomen (diaphragm and stomach) and limiting surgery to omentectomy and palliative small bowel/colon resection in case of obstruction. The reduction of tumor burden is extremely beneficial in this group of patients, although this strategy remains inferior with respect to complete resection [27]. In patients fit for surgery but with extensive disease, CRS-HIPEC is frequently associated with severe morbidity and a worse oncological outcome [28]. In this group of patients, a pilot study investigated a two-stage CRS-HIPEC approach in eight patients fitted for surgery with PCI > 20, low-grade histology requiring >3 small bowel anastomosis or >4 enterotomies [29]. The results in terms of morbidity and visceral sparing after staged second CRS-HIPEC were very encouraging, with 12.5% of severe complications and only three visceral resections required. More interestingly, the histopathological examination of all specimens and biopsies obtained at the time of the second surgery did not evidence signs of residual disease and fibrosis, suggesting a complete histologic response to first HIPEC. Moreover, after a median follow-up of 31.5 months, all patients were alive without recurrent disease. This result confirms the activity of HIPEC in PMP patients with low-grade PMP and opens the door to further therapeutic strategies in patients not fit for extensive surgery or extremely diffuse/unresectable disease.

### 4.3. Pressurized Intraperitoneal Aerosolized Chemotherapy (PIPAC)

PIPAC is an innovative mini-invasive technique introduced in 2011 to administer chemotherapy inside the peritoneal cavity with a normothermic high-pressure aerosol. PIPAC rationale relies upon aerosol physics behavior (ideal gas distribution law), with a claimed better intraperitoneal diffusion and higher tissue penetration (4 mm), compared to HIPEC, in which chemotherapeutics agents follow fluid distribution dynamics [30]. PIPAC is well-tolerated, as hospitalization is generally less than 3 days and adverse events (CTCAE > grade 2) are observed in 12–15% of cases [31]. The objective pathological response rate at repeated peritoneal biopsies was found to be 62–88%, 71–86% and 70–100% in ovarian, colorectal and gastric peritoneal metastases, respectively. Moreover, PIPAC improves symptoms control and quality of life compared to systemic chemotherapy alone [31]. PIPAC is currently proposed alone or in combination with systemic chemotherapy mainly in palliative settings with initial but encouraging data on survival. Clinical studies exploring the effectiveness of PIPAC plus systemic chemotherapy in neoadjuvant setting are ongoing [31].

The role of PIPAC in PMP has not been fully elucidated, given the disease rarity and the effectiveness of cytoreductive surgery combined with HIPEC, which remains the mainstay treatment. Nevertheless, in cases not eligible for CRS-HIPEC, PIPAC with Cisplatin/Doxorubicin may represent a valid therapeutic option. At present, although studies are limited to few cases, pressurized administration has showed good tolerability and there has been an objective pathological response also in PMP patients [32,33].

### 4.4. Intestinal Transplantation for End-Stage PMP

An extensive small bowel involvement (primary or recurrent) during the disease progression inevitably leads to intestinal failure, small bowel obstruction and abdominal wall fistulation. This condition is frequently fatal and associated with poor quality of life. Although traditionally contraindicated in peritoneal malignancy, home parenteral nutrition (HPN) might limit nutritional failure. HPN can provide long-term nutritional support for a median of 338 days (71–2198) in patients with low- and high-grade PMP [34], but it is not able to palliate obstruction symptoms and abdominal wall entero-cutaneous fistulas. In these cases, salvage cytoreductive surgery followed by small bowel/multivisceral transplant (SBMT) has been tested [35,36]. In a recent review, out of the eight patients treated with SBMT, three died in the post-operative period for intestinal fistula and graft versus host disease (GVHD), without rejection of the intestine. After a range of 6–36 months following SBMT, the remaining five patients were HPN-free and two of them disease-free, with an excellent quality of life [37]. Radical cytoreduction and SBMT could, thus, prolong life in selected patients with PMP with end-stage disease. However, long-term outcomes remain unknown and should be further evaluated. An accurate patient selection and a close collaboration between peritoneal cancer surgeons and the transplant team is mandatory in order to optimize the results of such a complex procedure.

## 5. Systemic Treatments

### 5.1. Perioperative Systemic Chemotherapy

The role of systemic chemotherapy (SC) for PMP is a relatively poorly investigated topic. In potentially resectable patients, neoadjuvant systemic chemotherapy offers the theoretical advantage to decrease the tumor burden and reduce surgery extension and at lower risk of complications. However, pre-operative systemic chemotherapy did not show any benefit in patients with either low- and high-grade histology, and may only delay CRS-HIPEC, leading to a detrimental effect on surgery. Previous studies on SC before CRS-HIPEC have shown a worse survival outcome in patients with peritoneal metastases with a high-grade histology, with or without signet ring cells [38]. Similarly, a substantial advantage in treating patients with SC after CRS-HIPEC was not observed. Although some selection biases (retrospective design, single institution experiences and different drug combinations) might have an altered result interpretation, the SC routinely offered in colorectal metastatic disease (5FU, Oxaliplatin, Irinotecan, anti-vascular epithelial growth factor (VEGF) and epidermal growth factor receptor (EGFR) inhibitors in various combinations) seems ineffective in patients with potentially resectable or optimally resected PMP, and further strategies should be considered to improve surgical results [10].

### 5.2. Palliative Systemic Chemotherapy

When surgery is not indicated due to comorbidities or for unresectable disease, SC is considered, with the main aim to avoid progression and control symptoms. In general, a relatively unresponsiveness and chemoresistance of PMP cells to systemic chemotherapy is reported, due to their low proliferation rate and the uncertain drug availability in the mucinous microenvironment of tumor nodules [39]. Moreover, tumor response is difficult to evaluate with standard radiological criteria, as PMP masses are mostly composed of mucin and it is unlikely to obtain a significant shrinkage even in case of full activity on tumoral cells. The results of SC showed a response rate ranging between 8–20%, median OS between 26–56 months, and 1-year OS rate of 84–91 [40,41,42,43,44] (Table 3).

Although the recent introduction of immunotherapy has shown promising results in colorectal cancer (CRC) patients, especially those with defective DNA mismatch repair system (dMMR) [45], this treatment has not yet been tested in PMP patients. An ongoing phase II study of Nivolumab and Ipilimumab is recruiting patients with metastatic mucinous colorectal and appendiceal tumors with proficient DNA MMR (ClinicalTrials.gov Identifier: NCT03693846).

### 5.3. Anti-Angiogenic Treatment 

In preclinical models of PMP, tumor tissues were found to be vascularized and enriched in relevant pro-angiogenic factors associated with VEGF signaling, and inhibitors of this pathway, such as bevacizumab, were able to interfere with tumor growth in murine PMP xenografts [46]. Similarly, intraperitoneal administration of bevacizumab in an orthotopic murine model of PMP is correlated with the normalization of tumor vascularity, confirming the potential activity of anti-VEGF target therapy in PMP [47]. Bevacizumab has been tested in human high-grade PMP with encouraging results [48,49,50]. Further studies are needed to clarify the potential role of these drugs in patients with unresectable PMP.

## 6. Prognostic Significance of Pathological Markers

Several molecular predictors, mainly measurable by immunohistochemistry (IHC) in PMP specimens, have been investigated (Table 4). A study conducted on 65 PMP disclosed that high EGFR and Ki67 score, in addition to aneuploidy, significantly predicted highly aggressive histological subtypes [51]. Moreover, Ki67 score was associated with poor survival in addition to aneuploidy, independently from PMP subtypes [51]. Overexpression of p53 in tumor samples was linked to poor survival in a cohort of 194 PMP patients, whereas *KRAS* mutation did not show a prognostic value in a smaller group of these patients [52]. dMMR and mucin staining were also analyzed for prognostic significance, in addition to Ki67 labeling index, altered p53 staining and clinical variables including grading, lymph node involvement, angiolymphatic and perineural invasion. While univariate analysis identified several potential prognostic factors, only histological grade remained an independent factor in multivariable analysis [53]. More recent studies were aimed at confirming the hypothesis that Ki67 quantification might have a prognostic significance in PMP. Indeed, Ki67 score was recently significantly associated with histological grade and survival, in addition to aberrant detection of p53, lymph node metastases and angiolymphatic invasion [54]. A cut-off value of 15% for Ki67 staining was found to subdivide high-grade PMP into two distinct groups, with a statistically significant difference in OS and disease-free survival [55]. Multicenter studies to identify universal cut-off values of potentially prognostic molecular factors are needed.

## 7. Novel Therapeutic Targets

Genomic, transcriptomic and protein analyses were conducted on small cohorts of PMP samples to define DNA, RNA and protein changes that might characterize PMP biology and highlight actionable targets for PMP therapy. However, PMP represents a challenge for the paucity of cellularity in many samples, which may impair the quality and the reliability of the obtained data. In fact, great variability in detection rates of mutations in PMP samples was observed. Differences in the amount of tumor cells in the analyzed samples as well as in the sensitivity of the used detection methods may account for data inconsistency.

### 7.1. Genomic and Transcriptomic Profile in PMP

Genetic alterations in *KRAS* and *GNAS* were demonstrated to contribute to an increased mucin production in PMP and CRC. In fact, knockdown of mutant *KRAS* with short hairpin RNA reduced MUC2 expression in mucinous CRC cell lines harboring this mutant oncoprotein, whereas wild type *KRAS*, HRAS and NRAS were not essential for MUC2 expression in the same cells [56]. Mutant *GNAS* was shown to determine a constitutive activation of the cAMP/PKA signaling pathway, which is involved in mucin overexpression [57]. It was reported that a PMP patient with a *GNAS* mutation, and refractory to several conventional chemotherapeutic agents, experienced a clinical benefit from trametinib, a MEK1/2 inhibitor [58], normally used in combination with dabrafenib in patients with BRAF-mutated melanoma. Nevertheless, studies performed on samples enriched in tumor cells identified exclusively wild type *KRAS* and *GNAS* in high-grade disseminated mucinous appendiceal tumors with a high percentage of signet ring cells [59], suggesting that mutations in these genes might not be fully relevant to PMP aggressiveness. 

Mutant *KRAS* acts through the synergistic engagement of MEK/ERK and PI3K/AKT pathways [56]. The combined blockade with MEK and PI3K inhibitors proved to overcome the emerging resistance to single inhibitors in a subcutaneous LS174T murine xenograft model and to efficiently lead to tumor shrinkage [56]. A dual pathway inhibition in patients with advanced tumors exhibited good efficiency but great toxicity [60,61]. Direct inhibitors of mutant *KRAS* have more recently been generated and have given encouraging results in vitro, in preclinical models and in a few treated patients [62,63], opening new possibilities in PMP treatment. Compared to CRC, the rate of *KRAS* mutation is doubled (up to 90%) and BRAF mutation is very rare [50]. These data suggest that VEGF represents a more efficient target for systemic treatment, as discussed above.

Exon-array analyses performed on PMP samples, an appendiceal mucinous tumor and two immortalized cell lines derived from primary appendiceal tumor tissue evidenced a differential expression of 61 genes compared to normal colonic epithelium, among which *SLC16A4*, *DSC3*, *ALDOB*, *EPHX4* and *ARHGAP24* were upregulated, while *MS4A12*, *TMIGD1* and *CASP5* were downregulated [64]. Whole-genome expression microarrays performed on tissue samples from 41 peritoneal metastases from appendiceal tumors and from CRC [65] showed three distinct profiles, two for PMP and one for CRC, with prognostic significance. In particular, low-grade appendiceal tumors were subdivided in two expression patterns with different clinical outcomes. This finding was further confirmed by a subsequent study, which defined a 139-gene cassette overexpressed in patients with disseminated low-grade appendiceal tumors with poor prognosis [66], thus suggesting actionable targets, in terms of single genes or activated pathways, for patients with more aggressive PMP.

### 7.2. Protein Expression in PMP

Protein expression analyses in PMP were mainly performed by IHC in concomitance with mutational profiling. IHC studies on tumor samples from 54 PMP patients showed intense staining for COX-2, EGFR, cMET, cKIT and platelet-derived growth factor receptor-α (PDGFR-α) in about 60–80% of the analyzed tumors [67]. A study conducted on 183 appendiceal adenocarcinoma, including 66 PMP, demonstrated overexpression of ERCC1, TOPO1, PTEN and MGMT [68]. A comprehensive analysis of appendiceal cancers, including 28 PMP, confirmed the expression, albeit at different percentages, of the proteins evidenced by the two previous studies [68,69], highlighting that protein analyses might give consistent data and also could suggest targets for supporting therapies, such as inhibitors of TOPO1 and EGFR and antagonists of PDGFR.

## 8. Mucin as a Therapeutic Target

### 8.1. Mucins in PMP

Mucins are high molecular weight glycoproteins physiologically produced by epithelial cells on apical surfaces for lubricating and protecting the epithelia of ducts and body cavities against bacteria, digestive enzymes, acids, toxic substances and mechanical damage [70]. A deregulated expression of mucins contributes to inflammation, tumorigenesis and metastasis of epithelial malignancies [70]. Knowledge of the pathways involved in coordinated regulation and an aberrant expression of multiple mucins during cancer development and progression might provide valuable targets for the development of effective antineoplastic treatments.

Human mucins have been classified into transmembrane and secreted forms. The transmembrane mucins have a single membrane-spanning region and form a protective mucous gel through their ectodomains. They selectively interact with various cellular and extracellular ligands and mediate signal transduction through the C-terminal cytoplasmic tail that functions as docking site for scaffolding proteins [71]. The secreted forms are subclassified in gel-forming and non-gel-forming mucins. Secreted mucins are characterized by a large size and high degree of glycosylation, and they are responsible for the viscoelastic properties of mucus. PMP is mainly characterized by the expression of secreted, gel-forming mucins (mainly MUC2, MUC5AC and MUC5B), which are claimed as responsible for the majority of morbidities and complications in patients, as well as for therapeutic resistance to systemic treatment [72].

### 8.2. Mucolytic Agents in PMP

The rationale for proposing mucolytic therapy in PMP is based on two principal aims: firstly, dissolving the incrementing amount of gel can decrease abdominal compression ameliorating quality of life; secondly, it could improve surgical performance and tumor response to intraperitoneal chemotherapy.

Among the mucus-dissolving agents used in PMP, N-acetylcysteine (NAC) and bromelain have been tested [73,74]. NAC is an acetylated derivative of L-cysteine and acts as a classic mucolytic drug. It decreases mucin viscosity by reducing the disulfide bonds, and also possesses anti-oxidant and anti-inflammatory properties [75]. Bromelain is a mixture of proteolytic and non-proteolytic enzymes extracted from the stems of the pineapple plant (*Ananas comosus*). Bromelain has several therapeutic benefits, among which immunomodulatory, anti-inflammatory and anti-neoplastic effects [76]. The activity of these two substances, alone or in combination, was extensively analyzed in the context of mucin-producing gastrointestinal cancer cell lines, mucinous ascites from PMP patients and preclinical models of PMP and PMCA [73,74]. They indeed showed significant mucolytic and antiproliferative effects, and these results were further confirmed in a phase I trial. This study demonstrated a manageable toxicity and an efficient in vivo mucus-dissolving activity of the two agents and prompted new investigations [77]. The percutaneous or laparoscopic route may be used to deliver mucolytic agents inside the peritoneal cavity before planned surgical treatment. In advanced unresectable PMP, the rationale of mucolysis is to reduce the disease burden in order to obtain symptoms relief and improve quality of life. Indeed, an ongoing phase I/phase II clinical trial analyzing intratumoral or intraperitoneal treatment/s with bromelain and NAC in patients affected by recurrent/inoperable mucinous peritoneal tumors, including PMP, should further prove the safety and efficacy of this therapeutic option for patients with inoperable mucinous tumors (ClinicalTrials.gov.: NCT03976973).

### 8.3. Targeting Expression of Mucin

**Figure 2:**
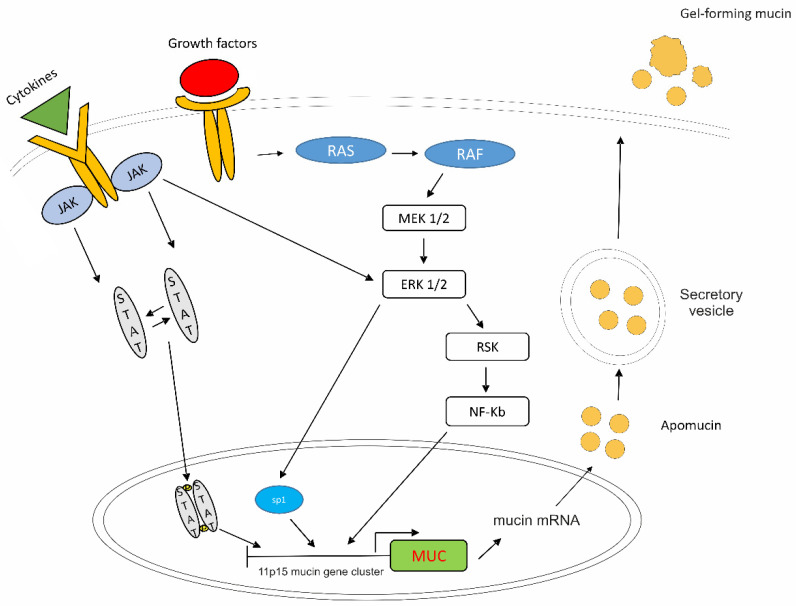
Main pathways involved in mucin expression in PMP. In the context of chronic inflammation, several factors, including pleiotropic cytokines, growth factors, hormones and LPS, may induce a phosphorylation cascade involving the Ras/Raf/MEK/ERK/RSK and JAK/STAT pathways, and their crosstalk, leading to SP1-, NF-Kb- and STAT-mediated activation of mucin promoters. The 11p15 mucin gene cluster contains four mucin genes, among which *MUC2*, *MUC5AC* and *MUC5B*, i.e., the genes mainly involved in mucin formation in PMP. Mucin mRNAs generate the core protein structures (apomucin) that are glycosylated, assembled and secreted through secretory vesicles. The three main mucins secreted by PMP are gel-forming mucins.

Several factors are involved in the triggering of mucin expression (Figure 2). The inflammatory microenvironment and different tumorigenic pathways have a prominent role in the upregulation of the mucin promoters, which could be used as biological tools for therapy. The 11p15 mucin gene cluster contains the four mucin genes *MUC2, MUC5AC*, *MUC5B* and *MUC6* [78]. Their promoters are modulated by several transcription factors, among which Sp1, Sp3, AP-1, NF-kB, ATF/CREB and RAR-α and -γ, and by epigenetic mechanisms [79,80,81]. Factors involved in mucin overexpression are proinflammatory and pleiotropic cytokines [interleukin-1beta (IL-1β), IL-4, IL-6, IL-9, IL-13 and tumor necrosis factor-alpha (TNF-α)], growth factors [epidermal growth factor (EGF) and transforming growth factor-α (TGF-α)], hormones (estrogen, T3, glucocorticoids), retinoids and bacterial and lipid mediators (LPS, PAF and PGE_2_) [80]. It was shown that LPS induces mucin overexpression in epithelial cells through a Src-dependent Ras/Mitogen-Activated Protein Kinase (MAPK)/pp90^rsk^ pathway leading to NF-kB activation [82]. Other intracellular signals that control mucin gene transcription are PKA-, PKC-, PKG- and Ca^2+^-dependent kinases and MAPK, the last one via EGFR [80].

Hypoxia-inducible factor-1α (HIF-1α) can upregulate the expression of factors associated with goblet cells, among which MUC2 and MUC5AC [83]. Using fresh tumor tissue from PMP patients and the mucinous LS174T CRC cells, a cell line that shows several features of goblet cells including high levels of MUC2 secretion, has shown that treatment with YC-1 and BAY 87─2243, two specific HIF-1α inhibitors, can significantly decrease MUC2 expression [84]. Moreover, by using an intraperitoneal xenograft mouse model of PMP, BAY 87─2243 treatment significantly prolonged animal survival compared to control mice, mainly inducing a decrease in mucin expression and in tumor burden. These data suggest that hypoxia targeting might represent a supporting treatment to reduce mucin production and tumor growth [84].

As reported above, MAPK signaling is the dominant upstream pathway that regulates *MUC2* gene expression [80,82,85]. PMP presents with variable percentage of *KRAS* mutations as well as intraperitoneal abundance of several factors involved in chronic inflammation, and these two conditions are implicated in the hyperactivation of MAPK signaling [86,87]. It was, therefore, investigated whether targeting MAPK might represent a strategy to reduce mucin production and possibly PMP growth. RDEA119 (BAY 86-9766) is a highly selective MAPK extracellular signal-regulated kinase (ERK) kinase 1/2 (MEK 1/2)-inhibitor exerting an antiproliferative effect in several xenograft models and in patients with advanced tumors [88]. In vitro, RDEA119 was found to efficiently inhibit *MUC2* transcription in mucin-producing LS174T CRC cells as well as in PMP tumor explants by reducing ERK1/2 phosphorylation [84]. Moreover, this MEK1/2 inhibitor was shown to exert a significant anti-neoplastic effect in preclinical PMP models, by decreasing mucinous tumor growth and increasing survival in a subcutaneous LS174T murine xenograft model as well as in an intraperitoneal xenograft model of passaged PMP tissue in nude mice [84].

## 9. Other Potential Therapeutic Strategies

### 9.1. JAK/STAT Pathway

IL-9 and its receptor (IL-9Rα) were shown to be more expressed in PMP than in CRC [89]. IL-9 is a multifunctional cytokine that seems to play a dual role in cancer development [90]. IL-9 activities are mediated by a specific receptor chain that forms a heterodimeric receptor with the common gamma chain (γ_c,_ also called IL2RG or CD132), involved in IL-2, -4, -7, -15 and -21 signaling [91]. IL-9Rα and γ_c_ associate with Janus kinase 1 (JAK1) and JAK3, respectively. Phosphorylated JAK1 and JAK3 then mediate the phosphorylation of receptor tyrosine residues. Phosphorylated tyrosine residues of IL-9Rα act as docking sites for the downstream signaling molecules, such as Signal Transducer and Activator of Transcription (STAT) transcription factors, insulin receptor substrate (IRS) and the adaptors of MAPK signaling pathways [90]. IL-9 was found to promote lymphomagenesis [92], but can exert opposite activities in solid tumors, being pro-tumorigenic or anti-neoplastic according to the tumor type [90]. Indeed, IL-9 may exert a pro-tumorigenic activity through the interaction with microRNAs (miRNAs). IL-9 treatment of pancreatic cancer cell lines was found to increase their proliferation and metastatic properties through the modulation of the miR-200a/β-catenin axis [93]. Moreover, miR-208b-5p was found to target the 3′ untranslated region of IL-9 and to inhibit the STAT3 pathway in non-small cell lung cancer cells [94], thus inhibiting their invasive and migratory capacities. IL-9 stimulation of CRC cells gave conflicting data, and proliferation was inhibited or triggered depending on the assayed cell line, Caco-2 and KM12 cells, respectively [95,96].

Targeting JAK/STAT may be useful in tumors in which this pathway is aberrantly activated, as in PMP. Knockdown of JAK1 or STAT3 using shRNAs significantly reduced tumor growth and ascites development in a preclinical NOD-SCID *IL2rγ^−/−^* (NSG) mouse model of peritoneal dissemination of ovarian cancer [97]. A selective JAK_1/2_ inhibitor (AZD1480) gave similar encouraging results in the same model, as well as in cell lines and murine models of other solid tumors. However, a phase I clinical trial of this small molecule in solid tumors evidenced neurotoxicity and ended its use in patients [97].

### 9.2. The Dual Role of Anti-Inflammatory Drugs

Anti-inflammatory drugs were found to counteract extracellular mucin production in vitro and in vivo. Specifically, dexamethasone significantly reduced basal and Na-butyrate-induced levels of *MUC2* expression, and celecoxib, a cyclooxygenase-2 (COX-2) inhibitor, significantly decreased Na-butyrate-triggered *MUC2* expression in LS174T cells [87]. Dexamethasone exerted a significant antineoplastic activity in a murine xenograft model in which LS174T cells were subcutaneously injected, as it induced decreased tumor volume and improved survival compared to control, PBS-treated mice [87].

Dexamethasone also significantly reduced mucinous tumor mass in a PMP xenograft model, decreasing not only MUC2 content but also the number of tumor cells, suggesting that this drug in PMP might exert a dual role as inhibitor of mucin production and cell proliferation [87].

### 9.3. Ubiquitin-Proteasome System (UPS)

Tumor cells amass misfolded proteins that need to be degraded, and therefore, they are more sensitive to UPS inhibitors compared to normal cells [98]. Bortezomib is a clinically approved proteasome inhibitor inducing apoptosis in several cancer cell lines and used to treat multiple myeloma [99,100]. Bortezomib has minimal effects on normal cells but also as single agent in some recurrent epithelial tumors and in some primary peritoneal carcinomatosis [101]. It was shown that the combination of MMC with bortezomib increased apoptosis of CRC cell lines and reduced intraperitoneal tumor growth, especially under hypoxic conditions, in a xenograft mouse model of peritoneal CRC carcinomatosis [102]. Another UPS inhibitor, phosphoric acid, 2,3-dihydro-1,1-dioxido-3-thienyl diphenyl ester (HRF-3), displayed promising cytotoxic properties in different cancer cell lines and in ex vivo tumor cells from patients affected by tumors of different origin [103]. However, tumor cells from 7 PMP patients displayed higher resistance to bortezomib compared to ex vivo CRC primary cell cultures and variable responses to HRF-3 [103], suggesting that more studies are needed to understand the relevance of UPS inhibitors as antineoplastic agents in PMP.

### 9.4. EpCAM

Epithelial cell adhesion molecule (EpCAM, CD326) is a type I transmembrane glycoprotein promoting cell-to-cell contact via interaction with different adhesion molecules (such as CD44 and Claudins) and through the modulation of adhesion strength mediated by E-Cadherin [104]. EpCAM is generally overexpressed in carcinomas, and was found to be relevant for the detection of circulating tumor cells (CTC) and for cancer stemness [104,105]. MOC31PE immunotoxin was developed by covalently linking the monoclonal antibody MOC31, targeting EpCAM, to *Pseudomonas* exotoxin A (PE), the most lethal exotoxin synthetized by the bacterium *Pseudomonas aeruginosa*. In vitro studies showed that, after binding to EpCAM-positive tumor cells, MOC31PE is internalized and PE is able to block protein synthesis and to trigger apoptosis, rapidly inducing cell death [106,107,108]. This immunotoxin was tested as single intraperitoneal injection, alone or in combination with MMC, in well-established preclinical models of PMP and PMCA, and showed a significant but variable antineoplastic effect, depending upon the analyzed model [109]. Treatment with MOC31PE of ex vivo mucinous tumor tissues, obtained from patients and from xenograft models, induced poly (ADP-ribose) polymerase (PARP) cleavage and protein synthesis inhibition, indicating classic cytotoxic mechanisms in mucinous neoplasms [109]. This immunotoxin was tested with MMC in patients with peritoneal metastasis from CRC and appendix cancer undergoing CRS-HIPEC, and administered intraperitoneally on the first postoperative day (ImmunoPeCa trial) [110]. This small-scale study showed that the immunotoxin was safe, well-tolerated and characterized by a low systemic uptake [111]. An MOC31PE-induced inflammatory reaction could contribute to cell death, but the mechanisms remain to be elucidated in future studies [111]. This trial showed an encouraging long-term outcome, as the estimated 3-year OS was 78% for all patients, and 72% for patients with less favorable clinical characteristics [112].

## 10. Conclusions

Cytoreductive surgery associated with HIPEC should be considered the gold standard of treatment of Pseudomyxoma peritonei. To maximize the results, a histopathological review, radiological staging and surgery should be centralized in experienced centers, as this allows to correctly treat all potentially curable patients and to avoid unnecessary treatments in unresectable or recurrent patients. Moreover, centralization and networking activity between referral centers set the ground for new therapeutic strategies, biobanking and translational research. The few studies available on the genomic, transcriptomic and protein profiles of PMP define peculiar research fields in PMP biology and highlight actionable targets for PMP therapy.

## Figures and Tables

**Table 1:** HIPEC regimens.

Drugs	Carrier Solution	Duration (min)
Oxaliplatin-Based		
Oxaliplatin (360–460 mg/m^2^) i.p. + 5FU (400 mg/m^2^) and LV (20 mg/m^2^) i.v.	5% dextrose solution, 2 L/m^2^	30
Oxaliplatin (200 mg/m^2^)	5% dextrose solution, 3 L	120
MMC-Based		
MMC (35 mg/m^2^) or 40 mg (Fixed Dose)	1.5% dextrose peritoneal dialysis solution, 3 L	90
MMC (10 mg/m^2^)	Sodium chloride solution, 0.9% 1 L	60
MMC (3.3 mg/L/m^2^) + Cisplatin (25 mg/m^2^/L)	Sodium chloride solution, 2.5 L/m^2^	60

Abbreviations: 5FU, 5-Fluorouracil; LV, Leucovorin; i.v., intravenously; i.p., intraperitoneally; MMC, Mitomycin C.

**Table 2:** Therapeutic approaches in PMP.

Treatment	Recommendations
CRS-HIPEC	All patients with a confirmed diagnosis of PMP should be treated in a referral center for CRS followed by HIPEC.
Palliative Surgery ± HIPEC	A debulking surgery with or without HIPEC provides disease control in high-risk patients or unresectable disease (primary or recurrent).
Systemic Chemotherapy	Adjuvant systemic chemotherapy should be considered in high-grade/signet ring PMP. In unresectable patients, palliative chemotherapy is effective in a minority of cases.
PIPAC	As palliative option within clinical studies.
Mucolytic Agents	As palliative option within clinical studies.
Small Bowel Transplant	In very selected patients with end-stage disease within clinical studies.

Abbreviation: PIPAC, pressurized intraperitoneal aerosolized chemotherapy.

**Table 3:** Systemic chemotherapy for unresectable PMP.

Regimen	N. of Patients	Median PFS (months)	Median OS (months)	RR (%)	DCR (%)
Capecitabine/5FU [40]	54	7.6	56	24	56
Capecitabine + MMC [41]	40	nr	84	8	42
Capecitabine + Bevacizumab [42]	15	8.2	91 ^a^	20	87
Capecitabine + Ciclophosphamide [43]	23	9.5	73.7 ^a^	4	87
FOLFOX6 [44]	8	8.0	26	20	65

^a^ Percentage at 1 year. Abbreviations: PFS, progression-free survival; OS, overall survival; RR, response rate; DCR, disease control rate; 5FU, 5-Fluorouracil; MMC, Mitomycin C; nr, not reported.

**Table 4:** Prognostic molecular parameters.

Analyzed Molecular Factors	N. of Patients	Association with Histological Grade	Association with Survival	Reference
COX-2, HER-2, EGFR, MUC2, Ki67	65	EGFR, Ki67	Ki67	[51]
p53/*KRAS* Mutations ^a^	194/64	p53	p53	[52]
dMMR, MUC ^b^, Ki67, p53	155	-	-	[53]
CEA, Ki67 and p53	141	Ki67, p53	Ki67, p53	[54]
Ki67, p53	117	Ki67	Ki67	[55]

^a^ All molecular markers were analyzed by IHC, except for *KRAS*, whose mutations in codons 12 and 13 were analyzed by shifted termination assay; ^b^ IHC staining for MUC1, MUC2, MUC5AC and MUC6.
